# Supplementary material for: Genome-wide CRISPR Screens in T Helper Cells Reveal Pervasive Crosstalk between Activation and Differentiation
Source: Cell. 2019 Feb 7;176(4):882–896.e18. doi: 10.1016/j.cell.2018.11.044 (PMC6370901; doi:10.1016/j.cell.2018.11.044)
Supplement: Data S2. Processed Data from All the Steps of the Analysis, Related to Figure 1 [file mmc2.zip › supplemental data/motif analysis/Bhlhe40_homer/homerResults/motif6.similar.html]

motif6

## Information for motif6

C
G
T
A
A
C
T
G
C
G
T
A
G
T
C
A
C
G
T
A
A
G
T
C
C
G
A
T
C
G
T
A
A
G
T
C
C
G
T
A
C
G
T
A
A
G
T
C
  
Reverse Opposite:  

A
C
T
G
A
C
G
T
A
C
G
T
A
C
T
G
A
C
G
T
C
G
T
A
C
T
A
G
A
C
G
T
A
C
G
T
G
A
C
T
A
G
T
C
A
C
G
T
  

|  |  |
| --- | --- |
| p-value: | 1e-14 |
| log p-value: | -3.354e+01 |
| Information Content per bp: | 1.898 |
| Number of Target Sequences with motif | 7.0 |
| Percentage of Target Sequences with motif | 0.58% |
| Number of Background Sequences with motif | 1.7 |
| Percentage of Background Sequences with motif | 0.00% |
| Average Position of motif in Targets | 99.6 +/- 58.6bp |
| Average Position of motif in Background | 96.2 +/- 47.6bp |
| Strand Bias (log2 ratio + to - strand density) | 0.4 |
| Multiplicity (# of sites on avg that occur together) | 1.00 |
| Motif File: | file (matrix) reverse opposite |

### Similar de novo motifs found

|  |  |  |  |  |  |  |  |
| --- | --- | --- | --- | --- | --- | --- | --- |
| Rank | Match Score | Redundant Motif | P-value | log P-value | % of Targets | % of Background | Motif file |
| 1 | 0.783 | T A C G C T G A C T G A T G C A A T G C T A G C G T C A A G T C T C G A G T C A | 1e-12 | -29.499610 | 3.00% | 0.65% | motif file (matrix) |
| 2 | 0.602 | A C G T C T A G G C A T C T A G C A T G A G C T G A C T C G T A | 1e-11 | -26.446540 | 6.75% | 2.86% | motif file (matrix) |
| 3 | 0.604 | C A T G G C T A A C T G C A G T G C T A C A T G A C G T T C G A A C T G C A G T | 1e-10 | -24.735335 | 1.67% | 0.23% | motif file (matrix) |
| 4 | 0.725 | C G T A A C T G A C G T A C G T A C T G A C G T C G T A A C T G | 1e-7 | -16.213281 | 1.58% | 0.35% | motif file (matrix) |
